# Author Correction: Cryo-EM structures of inhibitory antibodies complexed with arginase 1 provide insight into mechanism of action

**DOI:** 10.1038/s42003-021-02832-5

**Published:** 2021-11-15

**Authors:** Rachel L. Palte, Veronica Juan, Yacob Gomez-Llorente, Marc Andre Bailly, Kalyan Chakravarthy, Xun Chen, Daniel Cipriano, Ghassan N. Fayad, Laurence Fayadat-Dilman, Symon Gathiaka, Heiko Greb, Brian Hall, Mas Handa, Mark Hsieh, Esther Kofman, Heping Lin, J. Richard Miller, Nhung Nguyen, Jennifer O’Neil, Hussam Shaheen, Eric Sterner, Corey Strickland, Angie Sun, Shane Taremi, Giovanna Scapin

**Affiliations:** 1 Department of Discovery Chemistry, Merck & Co., Inc., Boston, MA USA; 2 Department of Discovery Biologics, Merck & Co., Inc., South San Francisco, CA USA; 3 Department of Discovery Chemistry, Merck & Co., Inc., Kenilworth, NJ USA; 4 Department of Discovery Biology, Merck & Co., Inc., Boston, MA USA; 5 Department of Preclinical Development, Merck & Co., Inc., Boston, MA USA; 6 Department of Discovery Biologics, Merck & Co., Inc., Boston, MA USA; 7 Department of Discovery Oncology, Merck & Co., Inc., Boston, MA USA; 8 Present Address: Ipsen Bioscience Inc., Cambridge, MA USA; 9 Present Address: Synthekine Inc., Menlo Park, CA USA; 10 Present Address: Xilio Therapeutics, Waltham, MA USA; 11 Present Address: Pandion Therapeutics, Cambridge, MA USA; 12 Present Address: NanoImaging Services, Woburn, MA USA

**Keywords:** Cryoelectron microscopy, Immunology

Correction to: *Communications Biology* 10.1038/s42003-021-02444-z, published online 29 July 2021.

In this article, the author Ghassan N. Fayad was inadvertently omitted from the author list. All authors agree with amending the author list. The author and affiliation lists have been updated to the following, with new additions shown in bold font:

*Rachel L. Palte^1^, Veronica Juan^2^, Yacob Gomez-Llorente^3^, Marc Andre Bailly^2^, Kalyan Chakravarthy^4,8^, Xun Chen^3^, Daniel Cipriano^2^, **Ghassan N. Fayad**^**5**^, Laurence Fayadat-Dilman^2^, Symon Gathiaka^1^, Heiko Greb^2,9^, Brian Hall^6^, Mas Handa^2^, Mark Hsieh^2^, Esther Kofman^2^, Heping Lin^6^, J. Richard Miller^4^, Nhung Nguyen^2^, Jennifer O’Neil^7,10^, Hussam Shaheen^6,11^, Eric Sterner^6^, Corey Strickland^3^, Angie Sun^6^, Shane Taremi^6^, Giovanna Scapin^3,12^

Affiliations

^1^Department of Discovery Chemistry, Merck & Co., Inc., Boston, MA, USA

^2^Department of Discovery Biologics, Merck & Co., Inc., South San Francisco, CA, USA

^3^Department of Discovery Chemistry, Merck & Co., Inc., Kenilworth, NJ, USA

^4^Department of Discovery Biology, Merck & Co., Inc., Boston, MA, USA

^5^ **Department of Preclinical Development, Merck & Co., Inc. Boston, MA, USA**

^6^Department of Discovery Biologics, Merck & Co., Inc., Boston, MA, USA

^7^Department of Discovery Oncology, Merck & Co., Inc., Boston, MA, USA

^8^Present address: Ipsen Bioscience Inc., Cambridge, MA, USA

^9^Present address: Synthekine Inc., Menlo Park, CA, USA

^10^Present address: Xilio Therapeutics, Waltham, MA, USA

^11^Present address: Pandion Therapeutics, Cambridge, MA, USA

^12^Present address: NanoImaging Services, Woburn, MA, USA

*corresponding author (rachel.kubiak@merck.com)

In addition, the Author Contributions section has been updated to include contributions from Ghassan N. Fayad, shown in bold font here:

R.L.P, Y.G.-L., S.G., C.S., G.S. **and G.N.F** contributed to the acquisition, analysis, or interpretation of the structural data. V.J., M.A.B., L.F.-D., M. Handa, E.K., X.C., J.R.M., N.N., J.O., H.S., K.C., S.T., D.C., M. Hsieh, and H.G. contributed to the acquisition, analysis or interpretation of the biological and biophysical data. V.J., L.F.-D., B.H., M. Handa, E.S., A.S., H.L., S.T. and H.S. contributed to or directed the design and production of protein and/or antibodies. All authors critically reviewed or revised the paper for the intellectual content and approved the final version.

The errors have been corrected in the HTML and PDF versions of the article.

