# Supplementary material for: Cryo-EM structures of inhibitory antibodies complexed with arginase 1 provide insight into mechanism of action
Source: Commun Biol. 2021 Jul 29;4:927. doi: 10.1038/s42003-021-02444-z (PMC8322407; doi:10.1038/s42003-021-02444-z)
Supplement: Supplementary file 2 — Supplementary Information [file 42003_2021_2444_MOESM2_ESM.pdf]

Cryo-EM structures of inhibitory antibodies complexed with Arginase 1 provide insight into mechanism of action.

*Rachel L. Palte[1], Veronica Juan[2], Yacob Gomez-Llorente[3], Marc Andre Bailly[2], Kalyan Chakravarthy[4†], Xun Chen[3], Daniel Cipriano[2], Laurence Fayadat-Dilman[2], Symon Gathiaka[1], Heiko Greb[2], Brian Hall[5], Mas Handa[2], Mark Hsieh[2], Esther Kofman[2], Heping Lin[5], J. Richard Miller[4], Nhung Nguyen[2], Jennifer O'Neil[6¤], Hussam Shaheen[5§], Eric Sterner[5], Corey Strickland[3], Angie Sun[5], Shane Taremi[5^], Giovanna Scapin[3±]

Affiliations

1 Department of Discovery Chemistry, Merck & Co., Inc., Boston, MA, USA
2 Department of Discovery Biologics, Merck & Co., Inc., South San Francisco, CA, USA
3 Department of Discovery Chemistry, Merck & Co., Inc., Kenilworth, NJ, USA
4 Department of Discovery Biology, Merck & Co., Inc., Boston, MA, USA
5 Department of Discovery Biologics, Merck & Co., Inc., Boston, MA, USA
6 Department of Discovery Oncology, Merck & Co., Inc., Boston, MA, USA
* Corresponding author (rachel.kubiak@merck.com)
† Present address: Ipsen Bioscience Inc., Cambridge, MA, USA
¤ Present address: Xilio Therapeutics, Waltham, MA, USA
§ Present address: Pandion Therapeutics – a wholly owned
^ Present address: 155 Brookline St, #9, Cambridge, MA, USA
± Present address: NanoImaging Services, Woburn, MA, USA

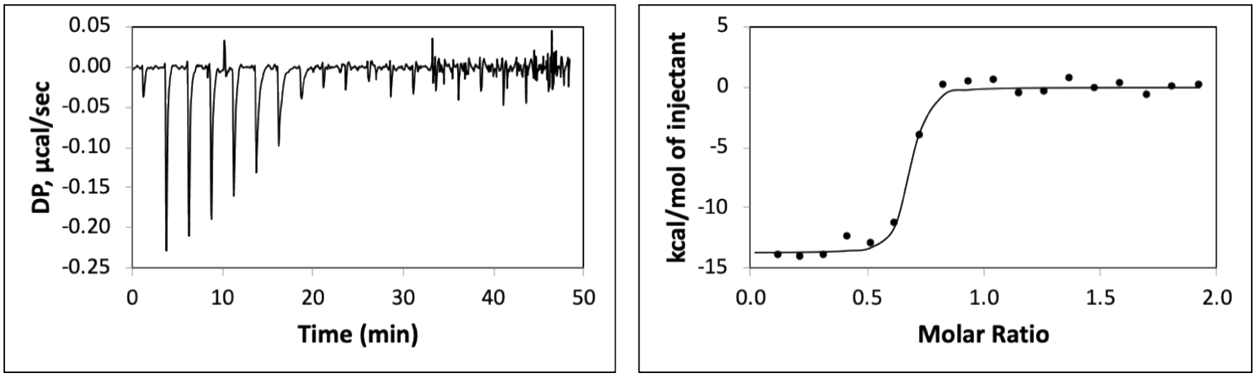

SI Figure 1. Isothermal Titration Calorimetry Assay results. The calculated stoichiometry n=0.637 is consistent with 2:3 ratio of [hArg1]:[mAb].

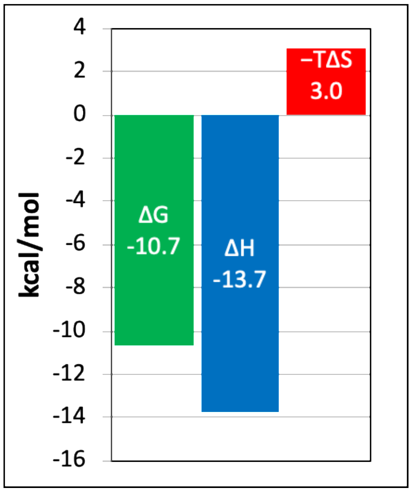

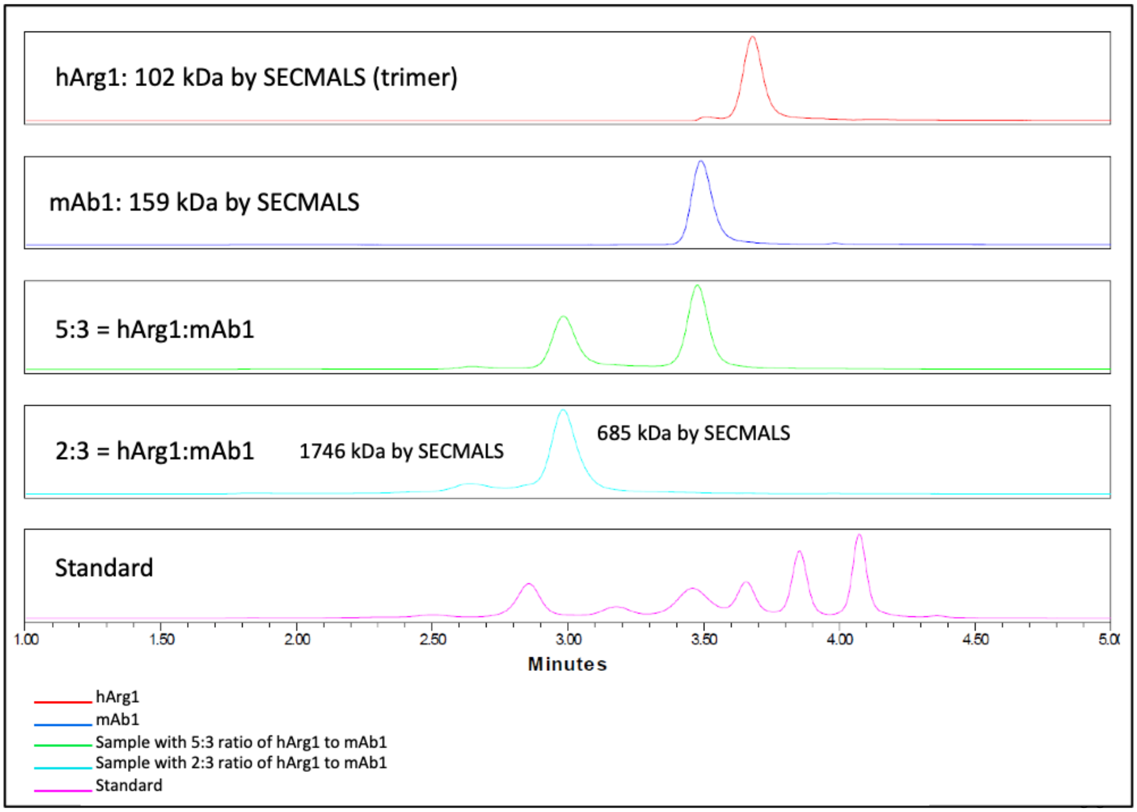

Name	Description	Calculated MW (Da)	MW SEC MALS (Da)
control	hArg1 Trimer	104,205	102,000
mAb1	Anti-hArg1 antibody	147,075	159,000

SI Figure 2. SECMAL results of hArg1 with mAb1 in two ratios. These results verify that the best fit for the hArg1:mAb1 ratio is 2:3.

SI Table 1: summary of cryo-EM data collection

Sample	mAb1	mAb2	mAb4	mAb3	mAb5
**Microscope**	Krios/EF	Krios/EF	Krios/EF	Krios/EF	Krios/EF
**Camera**	K2	K2	K2	K2	K2
**Pixel size (Å/pix)**	1.04	1.04	1.04	1.04	1.04
**Dose (e^-/Å)**	45.46	44.32	44.47	44.47	45.44
**#images**	1172	778	1166	1046	2048
**Defocus range (µm)**	-1.0 to -1.8	-1.2 to -2.0	-1.0 to -2.0	-1.0 to -2.0	-1.0 to -2.0
**Date**	30-May-2018	13-Jun-2018	15-Aug-2018	14-Aug-2018	10-Sep-2018

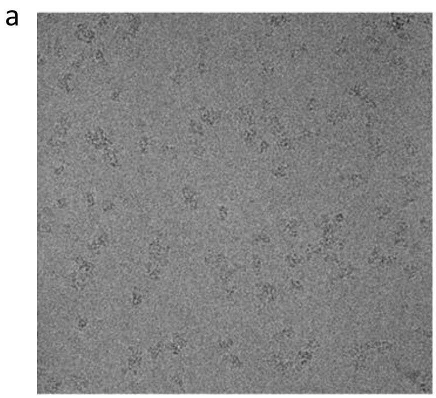

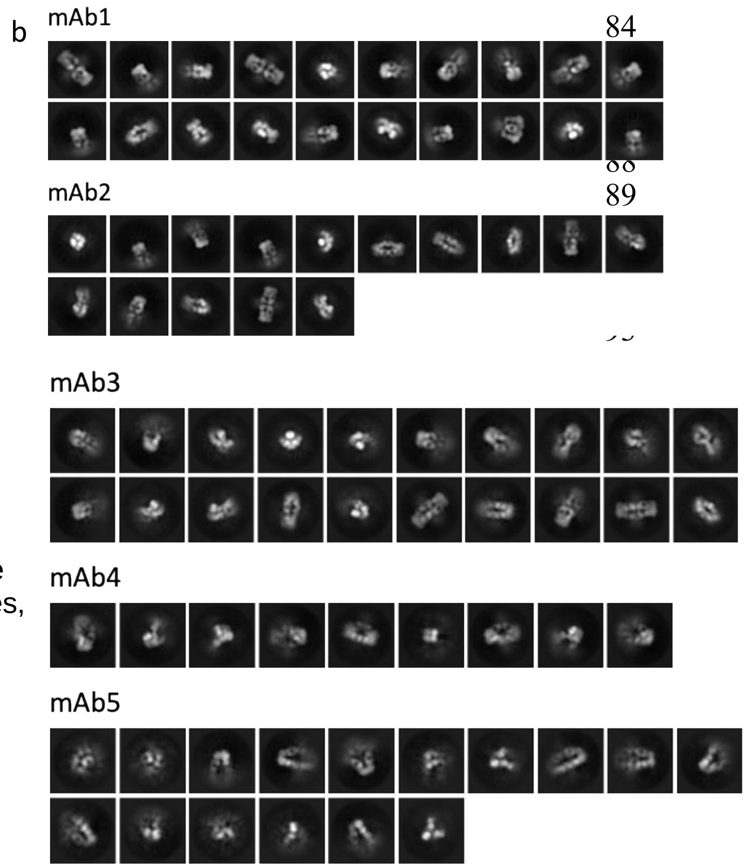

SI Figure 3. a) Representative micrograph of the hArg:mAb1 dataset. Micrographs from all datasets have a similar aspect. The bar represents 50 nm. **b)** 2D classes, selected after the 2D classification step for every dataset. These are representative of the particles used to generate the different reconstructions. Every class is comprised of 1,500 to 5,000 particles.

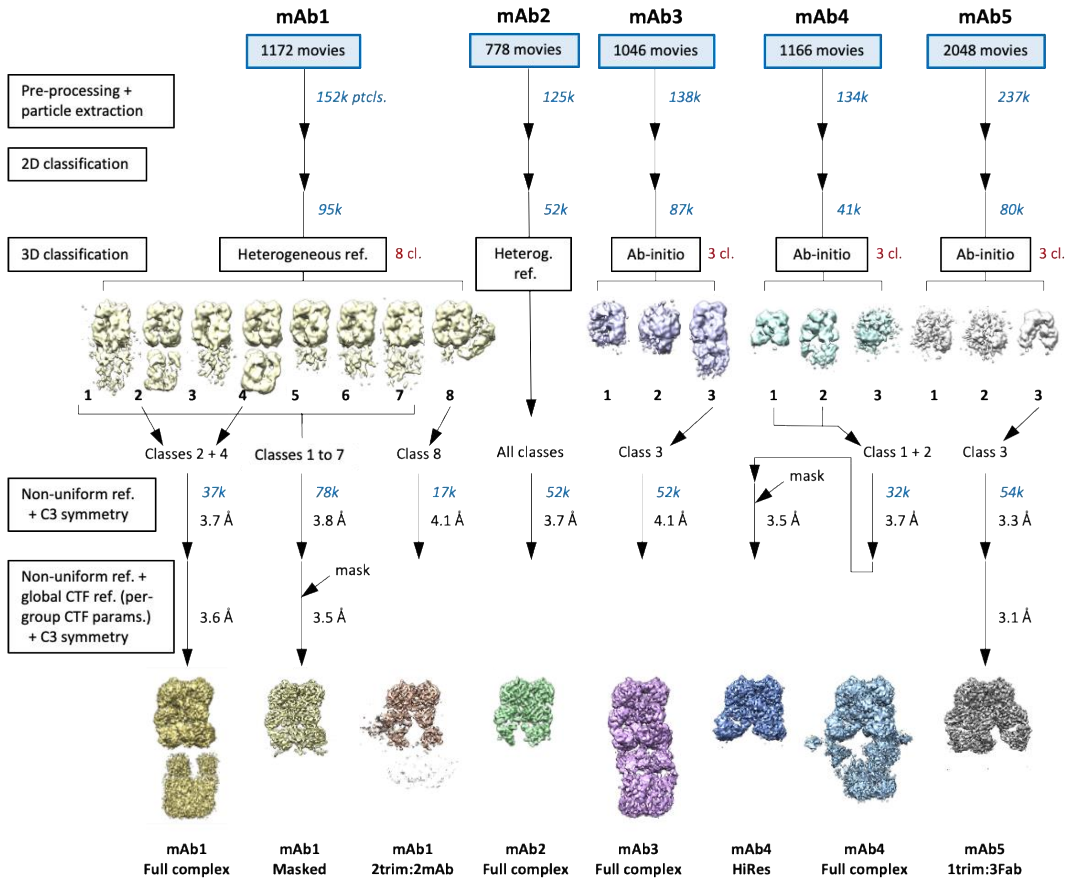

SI Figure 4. Summary of main processing steps for the cryo-EM datasets to generate each of the final reconstructions. The number of particles (in thousands) involved in every step of the processing is indicated in blue italic letters for the steps of particle extraction, particle selection after 2D classification, and 3D refinement. The number of classes for 3D classification step (either by heterogenous refinement or ab-initio) is indicated in red letters when necessary. Global CTF refinement successfully improved the final resolution in the cases of the “mAb1 full complex”, “mAb1 masked” and “mAb5 1 trimer:3 Fabs” reconstructions. The map for the “mAb2 full complex” is represented at a lower threshold, for clarity purposes. As a consequence, the second trimer of the map is not displayed in this representation. Additionally, some repetitive steps of the processing have been omitted from this summary for clarity purposes.

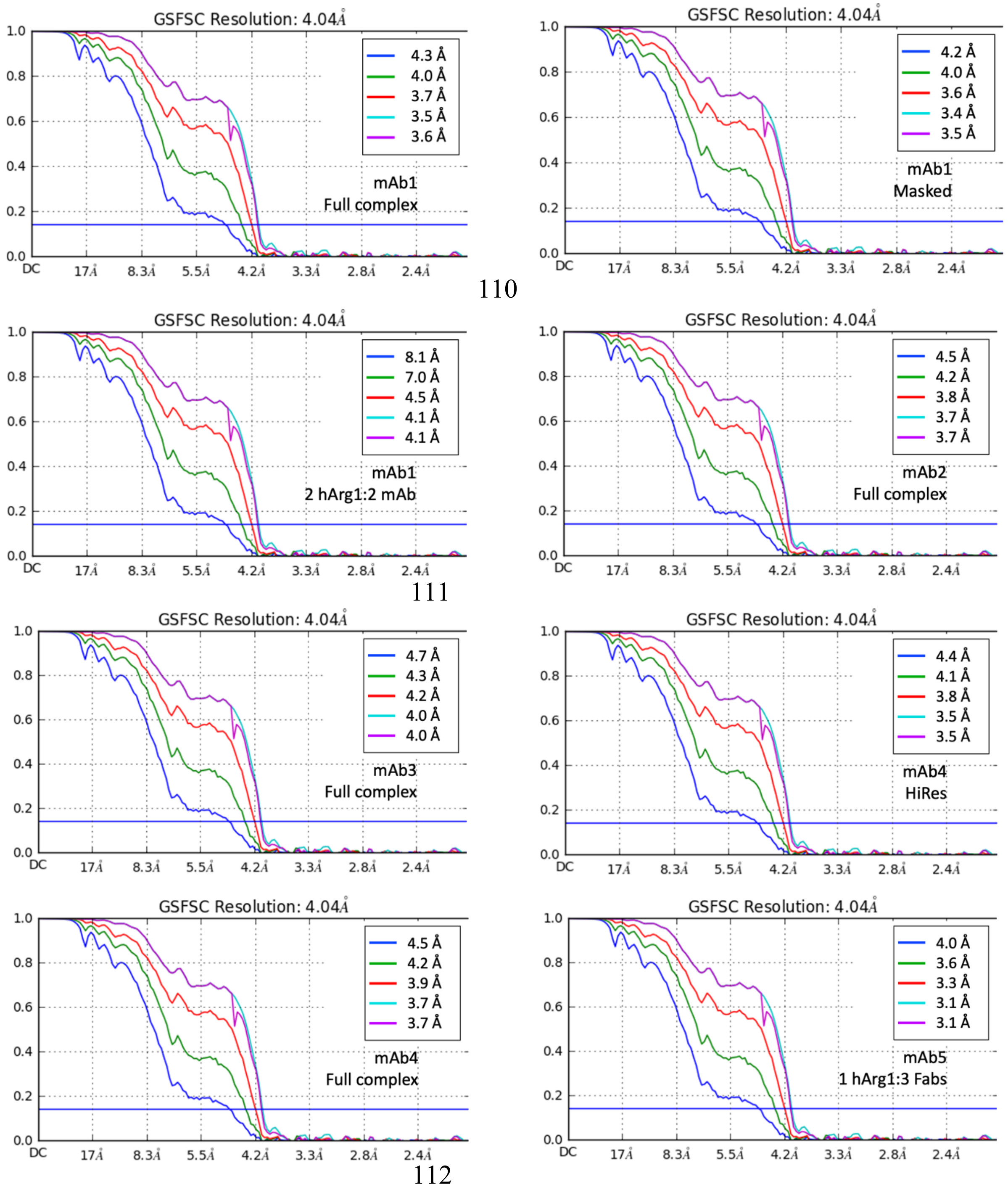

SI Figure 5. Gold-standard Fourier-shell correlation curves for each of the 8 reconstructions. The value of the “corrected” calculation plot at the 0.143 threshold is indicated. Line colors are: blue = no mask; green = spherical; red = loose, cyan = tight; and corrected = purple.

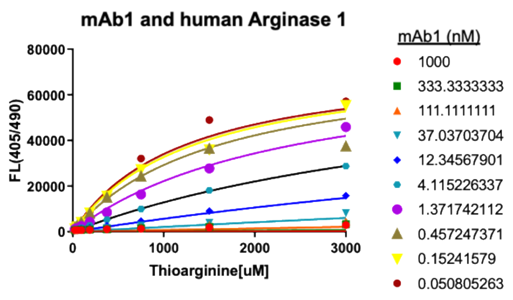

	Global (shared)
Comparison of Fits	
Simpler model	Competitive inhibition
Probability it is correct	99.99%
Alternative model	Noncompetitive inhibition
Probability it is correct	0.015%
Preferred model	Competitive inhibition
Difference in AICc	-17.62

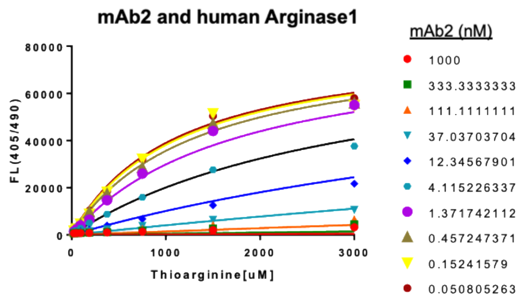

	Global (shared)
Comparison of Fits	
Simpler model	Competitive inhibition
Probability it is correct	99.98%
Alternative model	Noncompetitive inhibition
Probability it is correct	0.02%
Preferred model	Competitive inhibition
Difference in AICc	-17.18

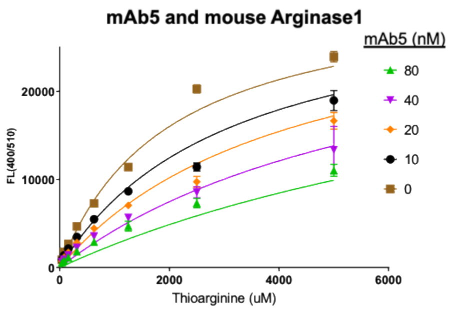

	Global (shared)
Comparison of Fits	
Simpler model	Competitive inhibition
Probability it is correct	>99.9%
Alternative model	Uncompetitive inhibition
Probability it is correct	<0.01%
Preferred model	Competitive inhibition
Difference in AICc	-34.74

SI Figure 6. Graphs showing the competitive inhibition profile of mAb1 and mAb2 against human Arg1 and mAb5 against mouse Arg1.

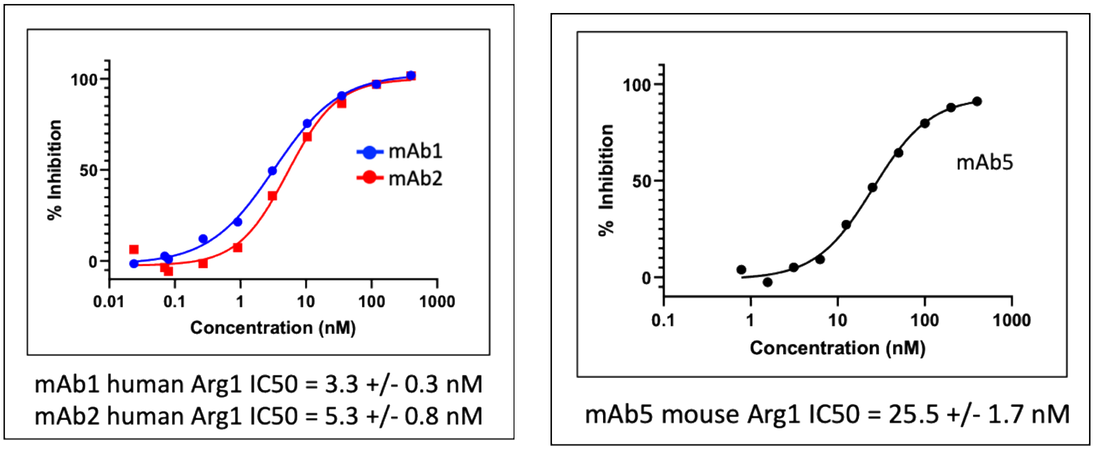

SI Figure 7. Dose response curves as determined by LCMS (mAb1 and mAb2) and TOGA (ThioOrnithine Generation Assay) (mAb5).

SI Table 2. Surface area, hydrogen bonds, and salt bridges between antibodies and hArg1.

antibody	Arg1 monomer	Antibody chain	Surface area (Å^2)	# Hydrogen bonds	salt bridges
mAb1-mAb3	MonA	HC	372	3	1
	MonB	LC	366	4	0
	MonB	HC	654	9	0
mAb4	MonA	HC	604	4	1
	MonA	LC	370	2	1
	MonB	LC	531	3	5
	MonB	HC	45	0	0
mAb5	MonA	HC	634	8	5
	MonA	LC	274	3	0

SA calcs done with PISA[11]

SI Table 3: Epitope interactions of mAb1, mAb2, and mAb3 with hArg1 paratope.

Heavy Chain	hArg1 monomer A
Tyr54	Lys39
Gly56	Thr290
Thr69	Pro286
Thr72, Asp73	Lys33
Thr74	Lys33, Ala34, Gly35, Glu38
Ser75	Arg32, Glu38
**Light Chain**	**hArg1 monomer B**
Ser28	Glu25
Tyr32	Ser16, Lys17, Asn69
Ser67	Asp57
Ser92	Pro20, Gly22
Leu93	Ser281
**Heavy Chain**	**hArg1 monomer B**
Tyr54	Asp181
Asn57*, Thr58	Lys284
Asn59”	Arg21
Tyr102	Pro20, Arg21
Gly103	Thr246
Tyr104	His126, Asp128, Asn130, Ser137, His141, Gly142, Asp183, Glu186
Arg105	Thr136, Asp183
Ser106	Ser137
Pro107	Thr136, Ser137
Tyr108	Lys68, Ser137, Asn139

All interactions shown here are within 4 Å

*Asn57 on mAb1 on mAb2; Glu57 on mAb3

“Asn59 on mAb1 on mAb2; His59 on mAb3

SI Table 4: Interactions between mAb4 epitope with hArg1 paratope.

Light Chain	hArg1 monomer A
Asn31	Glu42
Asp32	Lys39
Lys49	Glu38
Tyr50	Lys33, Ala34, Gly35, Glu38,
Gln53	Glu38, Lys41
Asn92	Lys39, Glu287, Thr290, Asn294
**Heavy Chain**	**hArg1 monomer A**
Thr30, Asp31	Glu26
Tyr33	Lys33, Pro280
Val50, Ser52	Pro286
Tyr54	Glu26, Ser281
Asn55	Gly283
Gly57	Lys284, Thr285, Pro286
Thr59	Pro286, Glu287
Asp99, Leu100	Lys33
Tyr101	Lys33, Ala34, Thr290, Val293
Tyr102	Glu38
**Light Chain**	**hArg1 monomer B**
Glu1	Arg21
Val3	Arg21
Arg24	Lys68
Ser26	Arg21
Gln27	Gly245, Pro247
Arg28	Asp181, Val182, Asp183, Pro184
Asp70	Thr136
Trp94	Lys284

All interactions shown here are within 4 Å

SI Table 5: Interactions between mAb5 epitope and hArg1 paratope.

Light Chain	hArg1 monomer A
Ser30	Pro54, Phe55
Asn31	Asp57
Tyr32	Phe55, Asp57
Ser49	Pro59
Ala50	Asp57, Pro59
Thr53	Pro59, Asn60
Tyr91	Asp57
**Heavy Chain**	**hArg1 monomer A**
Asp30, Asp31	Arg21
Asn52, Gly55	Ser281
Trp53	Arg21, Gly22
Asn54	Ser281, Leu282
Ser57	Glu26
Arg100	Pro59
Arg101	Lys17, Asp57, Ile58, Pro59
Arg102	Pro20, Arg21, Lys68
Gly103	Arg21, Gly22
Tyr105	Ser16, Lys17, Gln19, Pro20, Gly22, Glu25, Asn69
Gly106	Glu25

All interactions shown here are within 4 Å

SI Table 6. SPR data for mAb3 and mAb4 affinities for monomeric and trimeric hArg1

Human Arg1 - trimeric				
Antibody	ka (1/Ms)	kd (1/s)	KD (M)	STD KD (M)
mAb3	1.7×10^{6}	1.2×10^{-3}	**0.74×10^{-9}**	8.9×10^{-11}
mAb4	2.9×10^{5}	1.6×10^{-4}	**0.56×10^{-9}**	5.7×10^{-11}
**Human Arg1 - monomeric**				
Antibody	ka (1/Ms)	kd (1/s)	KD (M)	STD KD (M)
mAb3	**No binding**			
mAb4	3.9×10^{5}	7.6×10^{-3}	**20.0×10^{-9}**	6.8×10^{-11}

Supplementary note 1: Comparison of complex sizes and shapes

When considering the overall shape and size of the complexes (**SI Figure 8a**), it is clear that some of these differences are due to the varying backbone characteristics as described above. However, the epitope of the antibody is responsible for determining how and where the antibody binds to the hArg1 trimer which also plays a role in the antibody orientation and therefore the overall complex shape. In mAbs1-3 the epitope is at the very tip of the antibody (**SI Figure 8b**) with the HC accounting for the majority of the interactions with hArg1, resulting in the Fab binding almost perpendicular to the hArg1 trimer. Considering that cryoEM, ITC, and SEC-MALS data confirm the presence of the 2:3 complexes, the conformation of the mAbs binding to both the top and bottom halves of these complexes results in the antibodies taking on an almost T-shape appearance in which the angle of the backbone is ~150° degrees. This results in an elongated complex approximately 230 Å in length. In contrast, mAb4 has mostly LC interactions with the hArg1 trimer and binds in such a way that the Fab is splayed slightly outwards resulting in a shorter (~ 195 Å long) complex with a visibly smaller angle in the mAb backbone and a more rounded appearance. Lastly, mAb5 is unique among the five antibodies in that no second trimer is seen in electron density maps. The antibody interacts with hArg1 mainly through its HC, and with no specific orientation needed fr the mAbs to also bind a second trimer, the backbone angle and overall length of the complex is difficult to compare. The length of the top half of the complex can be measured at ~110-115 Å including the hArg1 trimer, and the complex seems to take on an even rounder appearance as compared to mAbs1-4.

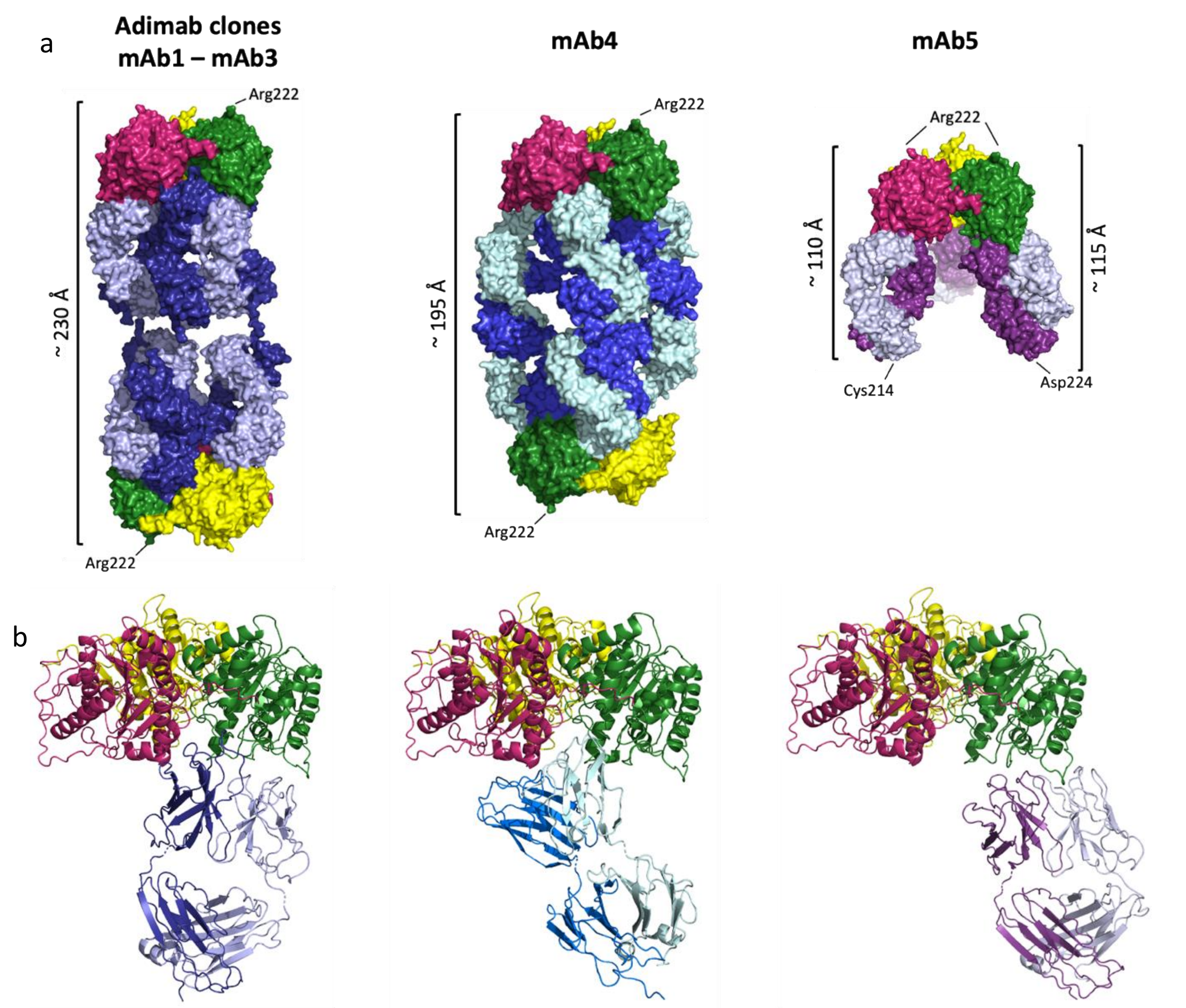

SI Figure 8. **Size and shape comparison of all three antibody clone types**. **a)** MAb1-mAb3 are approximately 230 Å in length when measuring between the C-alphas of the Arg222 residues in both the top and bottom monomer Bs (green) of hArg1. The length of mAb4 is also measured similarly between top and bottom Arg222 C-alphas and is shorter than mAbs1-3 at 195 Å. The mAb5 complex shows two separate measurements: one from the Arg222 C-alpha of one monomer to the terminal Cys214 residue of the LC (110 Å) and the other from the Arg222 C-alpha of the monomer to the terminal Asp224 residue of the HC (115 Å). **b)** The binding of one Fab and one hArg1 trimer are depicted in cartoon form and illustrate how antibodies bind to the hArg1 trimer and result in the overall shape difference of the large complexes.

Supplementary note 2: Ability of mAb1 to form a 2:2 complex

The immunoglobulin backbones differ between antibodies characterized here and seem to play a role in the formation of different structure classes identified microscopically. For instance, while mAbs1-3) all share identical epitope:paratope interactions, only mAb1 exhibited a 2:2 complex. In 2002[12] a fully intact human IgG including the hinge region confirmed that IgG hinges resemble "loose tethers," allowing the Fabs to rotate freely while still retaining a covalent link between the Fab and Fc domains. This flexible linkage, along with the specific antibody:antigen interactions, leads to the variance in Fab positioning and reach between the complexes.

In our study, the extended length of mAbs1-3 are approximately the same resulting in antibodies which share similar torsional rotations from the top trimer to the bottom. While mAb1 is built on a mouse IgG2a backbone, mAb2 is built on a mouse IgG1 backbone, which have been shown to be less flexible than the mouse IgG2a backbone hinge regions[13]. This enhanced flexibility in the IgG2a hinge region may be responsible for allowing the 2:2 complex to form with mAb1 but not mAb2. A possible scenario is that the 2:2 complex is formed first, followed by an opening up of first two mAb1s to permit a third mAb1 to bind to one hArg1 trimer and then eventually to the second hArg1 trimer. With a shorter and more rigid IgG1 backbone for mAb2, this extreme movement of the hinge is restricted and therefore only 2:3 complexes are seen. While it's difficult to compare the murine backbones of mAb1 and mAb2 directly to the human IgG4 backbone of mAb3, anisotropy decay studies showed that the mean time for decay of murine IgG2a was shorter than that of human IgG4, hinting at a more flexible murine IgG2a.[13] Therefore, although not directly assessed in this study, it suggests that the human IgG4 hinge region is more rigid than murine IgG2a, allowing only the 2:3 complexes to form. Further evidence supporting the hypothesis that the rigidity of the backbones prohibits the formation of the 2:2 complex is found in the lack of these complexes in all mAbs on the human IgG4 and mouse IgG1 backbones in this study.

Supplementary note 3: Antibody binding affinities to trimeric hArg1 and monomeric hArg1

Four of our five antibodies have interactions spanning across the hArg1 monomeric interfaces when hArg1 is present in the natural, trimeric form. SPR assays revealed the reduction or loss of binding potencies of the mAbs when hArg1 was forced into a monomeric state. The affinity matured mAb3 has numerous interactions with two monomers and we therefore hypothesized that mAb3 would have drastically reduced binding potency when hArg1 is monomerized. Indeed, while the binding of mAb3 to trimeric hArg1 was quite potent, measurable binding between mAb3 and monomeric hArg1 was completely lost (**SI Table 6**). When considering the surface area between hArg1 and mAb3, one monomer shares 372 $Å^2$ and 1 salt bridge with mAb3; the other monomer shares 1020 $Å^2$ of surface area but no salt bridges (**SI Table 2**). The nearly 75% reduction in shared surface area or interactions resulted in loss of all measurable mAb interaction.

MAb4 also binds across two monomers so a similar loss in potency was expected. However, the affinity of mAb4 for hArg1 differed only by ~36 fold when hArg1 was monomerized. Upon monomerization, mAb4 may have the ability to maintain binding to two separate hArg1 monomers. Within one hArg1 monomer, mAb4 shares 974 $Å^2$ of surface area and two salt bridges. With the other monomer, mAb4 shares only 576 $Å^2$ but has five salt bridges with hArg1. Despite having about 1.5-fold less surface area overlap in one of these mAb4:hArg1 pairs, the addition of several salt bridges may be enough to maintain binding. Therefore, although clearly losing potency upon monomerization, this ability to bind to two separate monomers of hArg1 may explain the conserved potency not seen with mAb3, though we do not have structural data to support this.

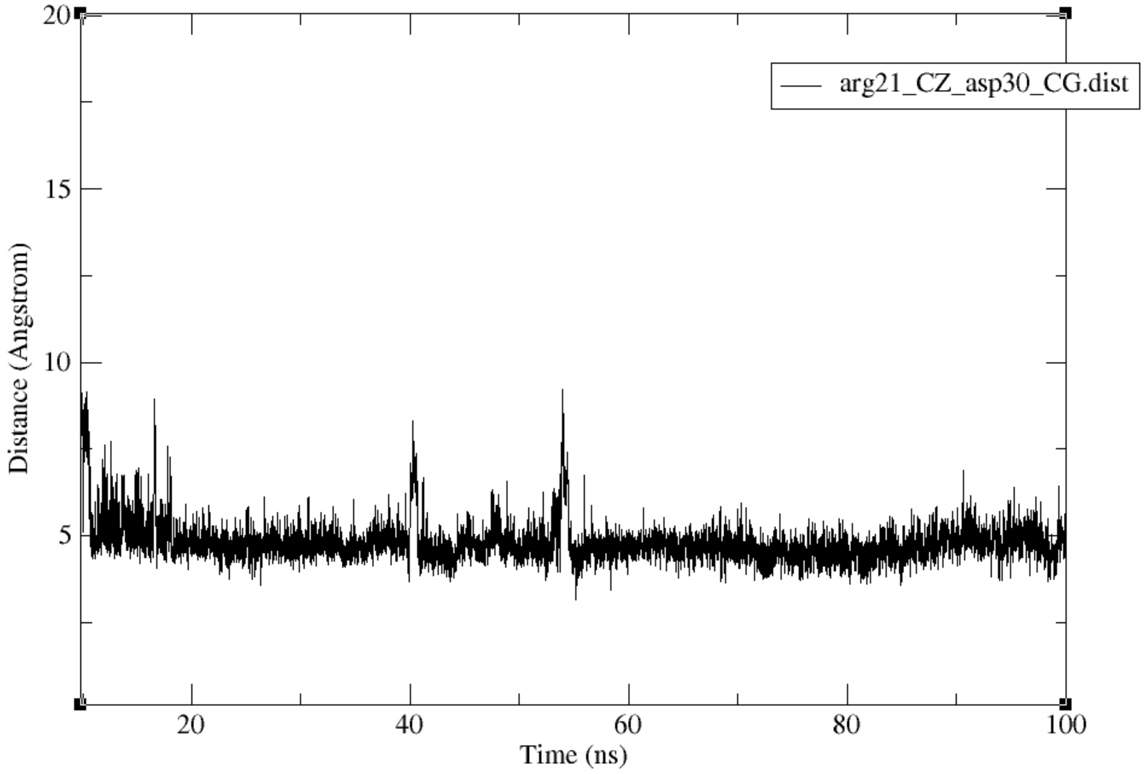

SI Figure 9: Distance (Å) over 100 ns of molecular dynamics simulations between hArg1's Arg21 (guanidino C atom CZ) and mAb5's Asp30 (carboxylate C atom CG).

Supplementary references

1. Sivasubramanian, A. *et al.* Broad epitope coverage of a human in vitro antibody library. *MAbs* (2017) doi:10.1080/19420862.2016.1246096.
2. Suloway, C. *et al.* Automated molecular microscopy: The new Leginon system. *J. Struct. Biol.* (2005) doi:10.1016/j.jsb.2005.03.010.
3. Zheng, S. Q. *et al.* MotionCor2: Anisotropic correction of beam-induced motion for improved cryo-electron microscopy. *Nature Methods* (2017) doi:10.1038/nmeth.4193.
4. Rohou, A. & Grigorieff, N. CTFFIND4: Fast and accurate defocus estimation from electron micrographs. *J. Struct. Biol.* (2015) doi:10.1016/j.jsb.2015.08.008.
5. Lander, G. C. *et al.* Appion: An integrated, database-driven pipeline to facilitate EM image processing. *J. Struct. Biol.* (2009) doi:10.1016/j.jsb.2009.01.002.
6. Punjani, A., Rubinstein, J. L., Fleet, D. J. & Brubaker, M. A. CryoSPARC: Algorithms for rapid unsupervised cryo-EM structure determination. *Nat. Methods* (2017) doi:10.1038/nmeth.4169.
7. Pettersen, E. F. *et al.* UCSF Chimera - A visualization system for exploratory research and analysis. *J. Comput. Chem.* (2004) doi:10.1002/jcc.20084.
8. Mitcheltree, M. J. *et al.* Discovery and Optimization of Rationally Designed Bicyclic Inhibitors of Human Arginase to Enhance Cancer Immunotherapy. *ACS Med. Chem. Lett.* **11**, 582–588 (2020).
9. Emsley, P. & Cowtan, K. Coot: Model-building tools for molecular graphics. *Acta Crystallogr. Sect. D Biol. Crystallogr.* (2004) doi:10.1107/S0907444904019158.

10. Afonine, P. V, Headd, J. J., Terwilliger, T. C. & Adams, P. D. New tool: phenix real space refine. *Comput. Crystallogr. Newsl.* (2013).
11. Krissinel, E. & Henrick, K. Inference of Macromolecular Assemblies from Crystalline State. *J. Mol. Biol.* **372**, 774–797 (2007).
12. Saphire, E. O. *et al.* Contrasting IgG structures reveal extreme asymmetry and flexibility. *J. Mol. Biol.* **319**, 9–18 (2002).
13. Dangl, J. L. *et al.* Segmental flexibility and complement fixation of genetically engineered chimeric human, rabbit and mouse antibodies. *EMBO J.* **7**, 1989–1994 (1988).
14. Jorgensen, W. L., Chandrasekhar, J., Madura, J. D., Impey, R. W. & Klein, M. L. Comparison of simple potential functions for simulating liquid water. *J. Chem. Phys.* (1983) doi:10.1063/1.445869.
15. Wang, L. P. *et al.* Building a More Predictive Protein Force Field: A Systematic and Reproducible Route to AMBER-FB15. *J. Phys. Chem. B* (2017) doi:10.1021/acs.jpcb.7b02320.
16. Roe, D. R. & Cheatham, T. E. PTRAJ and CPPTRAJ: Software for processing and analysis of molecular dynamics trajectory data. *J. Chem. Theory Comput.* (2013) doi:10.1021/ct400341p.
