# Supplementary material for: Cryo-EM structures of inhibitory antibodies complexed with arginase 1 provide insight into mechanism of action
Source: Commun Biol. 2021 Jul 29;4:927. doi: 10.1038/s42003-021-02444-z (PMC8322407; doi:10.1038/s42003-021-02444-z)
Supplement: Supplementary file 3 — Reporting Summary [file 42003_2021_2444_MOESM3_ESM.pdf]

nature research

Corresponding author(s): Rachel L. Palte

Last updated by author(s): May 20, 2021

Reporting Summary

Nature Research wishes to improve the reproducibility of the work that we publish. This form provides structure for consistency and transparency in reporting. For further information on Nature Research policies, see our Editorial Policies and the Editorial Policy Checklist.

Statistics

For all statistical analyses, confirm that the following items are present in the figure legend, table legend, main text, or Methods section.

n/a	Confirmed	
[ ]	[x]	The exact sample size (*n*) for each experimental group/condition, given as a discrete number and unit of measurement
[ ]	[x]	A statement on whether measurements were taken from distinct samples or whether the same sample was measured repeatedly
[x]	[ ]	The statistical test(s) used AND whether they are one- or two-sided<br>*Only common tests should be described solely by name; describe more complex techniques in the Methods section.*
[x]	[ ]	A description of all covariates tested
[x]	[ ]	A description of any assumptions or corrections, such as tests of normality and adjustment for multiple comparisons
[x]	[ ]	A full description of the statistical parameters including central tendency (e.g. means) or other basic estimates (e.g. regression coefficient) AND variation (e.g. standard deviation) or associated estimates of uncertainty (e.g. confidence intervals)
[x]	[ ]	For null hypothesis testing, the test statistic (e.g. *F*, *t*, *r*) with confidence intervals, effect sizes, degrees of freedom and *P* value noted<br>*Give P values as exact values whenever suitable.*
[x]	[ ]	For Bayesian analysis, information on the choice of priors and Markov chain Monte Carlo settings
[x]	[ ]	For hierarchical and complex designs, identification of the appropriate level for tests and full reporting of outcomes
[x]	[ ]	Estimates of effect sizes (e.g. Cohen's *d*, Pearson's *r*), indicating how they were calculated

Our web collection on statistics for biologists contains articles on many of the points above.

Software and code

Policy information about availability of computer code

Data collection	Data collection: 1) Data collection: Leginon (a software to operate electron microscopes and collect data; Suloway, C. et al. Automated molecular microscopy: The new Leginon system. J. Struct. Biol. (2005) doi:10.1016/j.jsb.2005.03.010).
Data analysis	2) Data pre-processing: MotionCor2 (a software to align frames and calculate motion corrected micrograph, Zheng, S. Q. et al. MotionCor2: Anisotropic correction of beam-induced motion for improved cryo-electron microscopy. Nature Methods (2017) doi:10.1038/nmeth.4193.) and CTFFIND-4 (a software to estimate micrograph CTF, Rohou, A. & Grigorieff, N. CTFFIND4: Fast and accurate defocus estimation from electron micrographs. J. Struct. Biol. (2015) doi:10.1016/j.jsb.2015.08.008.) 3) Data processing: Cryosparc (a software to calculate and refine electron microscopy volumes and maps (Punjani, A., Rubinstein, J. L., Fleet, D. J. & Brubaker, M. A. CryoSPARC: Algorithms for rapid unsupervised cryo-EM structure determination. Nat. Methods (2017) doi:10.1038/nmeth.4169.).<br>Data Analysis: Chimera (3D object visualization software, Pettersen, E. F. et al. UCSF Chimera - A visualization system for exploratory research and analysis. J. Comput. Chem. (2004) doi:10.1002/jcc.20084). COOT (3D structures building and visualization software, Emsley, P. & Cowtan, K. Coot: Model-building tools for molecular graphics. Acta Crystallogr. Sect. D Biol. Crystallogr. (2004) doi:10.1107/S0907444904019158). PHENIX (3D structure refinement software, Afonine, P. V, Headd, J. J., Terwilliger, T. C. & Adams, P. D. New tool: phenix real space refine. Comput. Crystallogr. Newsl. (2013).)

For manuscripts utilizing custom algorithms or software that are central to the research but not yet described in published literature, software must be made available to editors and reviewers. We strongly encourage code deposition in a community repository (e.g. GitHub). See the Nature Research guidelines for submitting code & software for further information.

Data

Policy information about availability of data

All manuscripts must include a data availability statement. This statement should provide the following information, where applicable:

- Accession codes, unique identifiers, or web links for publicly available datasets
- A list of figures that have associated raw data
- A description of any restrictions on data availability

3D structures and cryo-EM maps have been deposited in the PDB and EMDB. PDB accession codes are 7LEX, 7LEY, 7LEZ, 7LF0, 7LF1 and 7LF2. EMDB accession codes are: EMD-23293, EMD-23294, EMD-23295, EMD-23296, EMD-23297, EMD-23298

Field-specific reporting

Please select the one below that is the best fit for your research. If you are not sure, read the appropriate sections before making your selection.

☒ Life sciences ☐ Behavioural & social sciences ☐ Ecological, evolutionary & environmental sciences

For a reference copy of the document with all sections, see nature.com/documents/nr-reporting-summary-flat.pdf

Life sciences study design

All studies must disclose on these points even when the disclosure is negative.

Sample size	N/A
Data exclusions	N/A
Replication	N/A
Randomization	N/A
Blinding	N/A

Reporting for specific materials, systems and methods

We require information from authors about some types of materials, experimental systems and methods used in many studies. Here, indicate whether each material, system or method listed is relevant to your study. If you are not sure if a list item applies to your research, read the appropriate section before selecting a response.

Materials & experimental systems

n/a	Involved in the study	
☐	☒	Antibodies
☐	☒	Eukaryotic cell lines
☒	☐	Palaeontology and archaeology
☒	☐	Animals and other organisms
☒	☐	Human research participants
☒	☐	Clinical data
☒	☐	Dual use research of concern

Methods

n/a	Involved in the study	
☒	☐	ChIP-seq
☒	☐	Flow cytometry
☒	☐	MRI-based neuroimaging

Antibodies

Antibodies used	mAbs used were generated internally
Validation	N/A

Eukaryotic cell lines

Policy information about cell lines

Cell line source(s)	Gibco (Thermofisher)
Authentication	provided by supplier

Mycoplasma contamination	confirmed negative from supplier and internally
Commonly misidentified lines (See ICLAC register)	*Name any commonly misidentified cell lines used in the study and provide a rationale for their use.*
